# Cannabidiol Activates Integrated Stress Response Signaling and Immune Trafficking Programs in an A375 Melanoma–Jurkat T Cell Coculture Model: A Multi-Omics Analysis

**DOI:** 10.1021/acsomega.6c01965

**Published:** 2026-06-15

**Authors:** Ni Deng, Huifang Li, Ang Cai, Christopher L. Hemme, Jonghae Youn, Ryan Janis, Matthew D. Stone, Hang Ma, Chang Liu

**Affiliations:** † Proteomics Facility, 4260 College of Pharmacy, University of Rhode Island, Kingston, Rhode Island 02881, United States; ‡ Department of Biomedical and Pharmaceutical Sciences, College of Pharmacy, University of Rhode Island, Kingston, Rhode Island 02881, United States; § Rhode Island IDeA Network of Biomedical Research Excellence (RI-INBRE), Kingston, Rhode Island 02881, United States; ∥ 266634 Sciex, Redwood, California 94065, United States

## Abstract

Cannabidiol (CBD) is a nonpsychoactive cannabinoid with emerging anticancer and immunomodulatory properties; however, its systems-level mechanisms in tumor-associated immune cells remain incompletely defined. Here, we investigated CBD in a melanoma–T cell coculture model using integrated transcriptomic and proteomic analyses. At a subcytotoxic concentration (10 μM), CBD selectively induced apoptosis in melanoma while preserving T-cell viability and enhancing IL-2 secretion. RNA sequencing revealed coordinated activation of stress-adaptive, immune activation, and trafficking programs, including modulation of T-cell receptor signaling and cytokine networks. Data-independent acquisition proteomics identified activation of eukaryotic initiation factor 2 (EIF2) signaling, a central node of the integrated stress response (ISR) linking redox and endoplasmic reticulum stress to translational control. Multiomics integration converged on immune cell trafficking as a consistent outcome, with upregulation of ICAM1, ITGB1, and associated adhesion-related proteins. These findings suggest ISR-dependent translational reprogramming as a putative mechanistic axis by which CBD reshapes T-cell function in the melanoma microenvironment. Our study provides pharmacological insight into how CBD modulates tumor–immune interactions and suggests potential utility as an adjunct immunomodulatory agent in melanoma.

## Graphical abstract

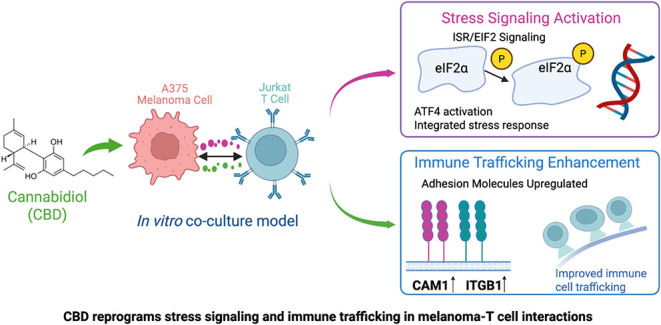

## 1 Introduction

Immune checkpoint inhibitors (ICIs), including PD-1 and CTLA-4 blockade, have transformed melanoma therapy; however, response rates remain limited and resistance frequently develops.^1,2^ Tumor-associated oxidative stress and translational reprogramming within T cells contribute to immune dysfunction and therapeutic failure.^3,4^ Pharmacological agents capable of modulating stress-adaptive signaling without compromising T-cell viability may enhance tumor–immune engagement and improve therapeutic responses. Therefore, identifying small molecules that engage conserved stress-response pathways in immune cells represents a translationally relevant strategy in melanoma. Redox signaling and cellular stress responses are fundamental regulators of cellular homeostasis and adaptive function across diverse biological systems.^5,6^ Reactive oxygen species (ROS) and redox-sensitive signaling pathways shape T-cell activation, differentiation, migration, and survival, while excessive oxidative or endoplasmic reticulum (ER) stress can suppress immune function or redirect immune responses.^7–9^ In cancer, dysregulated redox homeostasis contributes to tumor progression and immune evasion;^9,10^ thus, it is critical to understand how redox and stress-adaptive pathways govern tumor–immune interactions. Melanoma represents a clinically relevant model in which redox regulation and immune function are tightly intertwined.^11,12^ Increasing evidence suggests that redox imbalance and stress signaling play crucial roles in shaping these immune phenotypes,^13,14^ highlighting the need for mechanistic studies that integrate redox biology with tumor–immune crosstalk.

Plant-derived redox-active metabolites have emerged as important modulators of these stress-adaptive pathways, acting through conserved molecular nodes that integrate oxidative stress with cellular signaling. Cannabidiol (CBD) is a nonpsychoactive phytochemical that has attracted growing attention as a redox-active compound with antioxidant, cytoprotective, and anticancer properties.^15–17^ Apart from its direct effects on tumor cells, CBD has been reported to modulate inflammatory signaling, oxidative stress responses, and cell death pathways, including ferroptosis.^18–20^ Despite these advances, the mechanisms by which CBD reshapes stress-adaptive signaling in immune cells, particularly under conditions of tumor-associated stress, remain poorly defined at the systems level.

A central node linking redox imbalance to immune regulation is the integrated stress response (ISR), a conserved signaling network that coordinates translational regulation and cellular adaptation under oxidative and ER stress.^21,22^ In T cells, ISR activation has been shown to influence activation thresholds, effector differentiation, and migratory behavior, which suggests that stress-responsive translational control is a key regulator of immune function.^21,23^ In addition to intracellular signaling, redox and stress pathways also regulate immune cell trafficking, a process essential for effective antitumor immunity.^14,24^ Given that many phytochemicals exert their bioactivities through redox-sensitive stress pathways, ISR activation represents a plausible and underexplored mechanism underlying CBD’s immunomodulatory effects.

Thus, we employed a melanoma–T cell coculture model combined with integrated transcriptomic and proteomic analyses to investigate how CBD-driven redox and stress responses reshape T-cell function in the presence of tumor cells. Using a multiomics framework, we set out to (1) define stress-responsive signaling pathways engaged by CBD in T cells, (2) elucidate the role of ISR-related translational control, and (3) identify downstream functional consequences on immune activation and trafficking (Figure 1). This work aimed to provide mechanistic insight into how cannabidiol engages conserved stress-response networks to regulate immune function in a tumor-associated environment.

**Figure 1:**
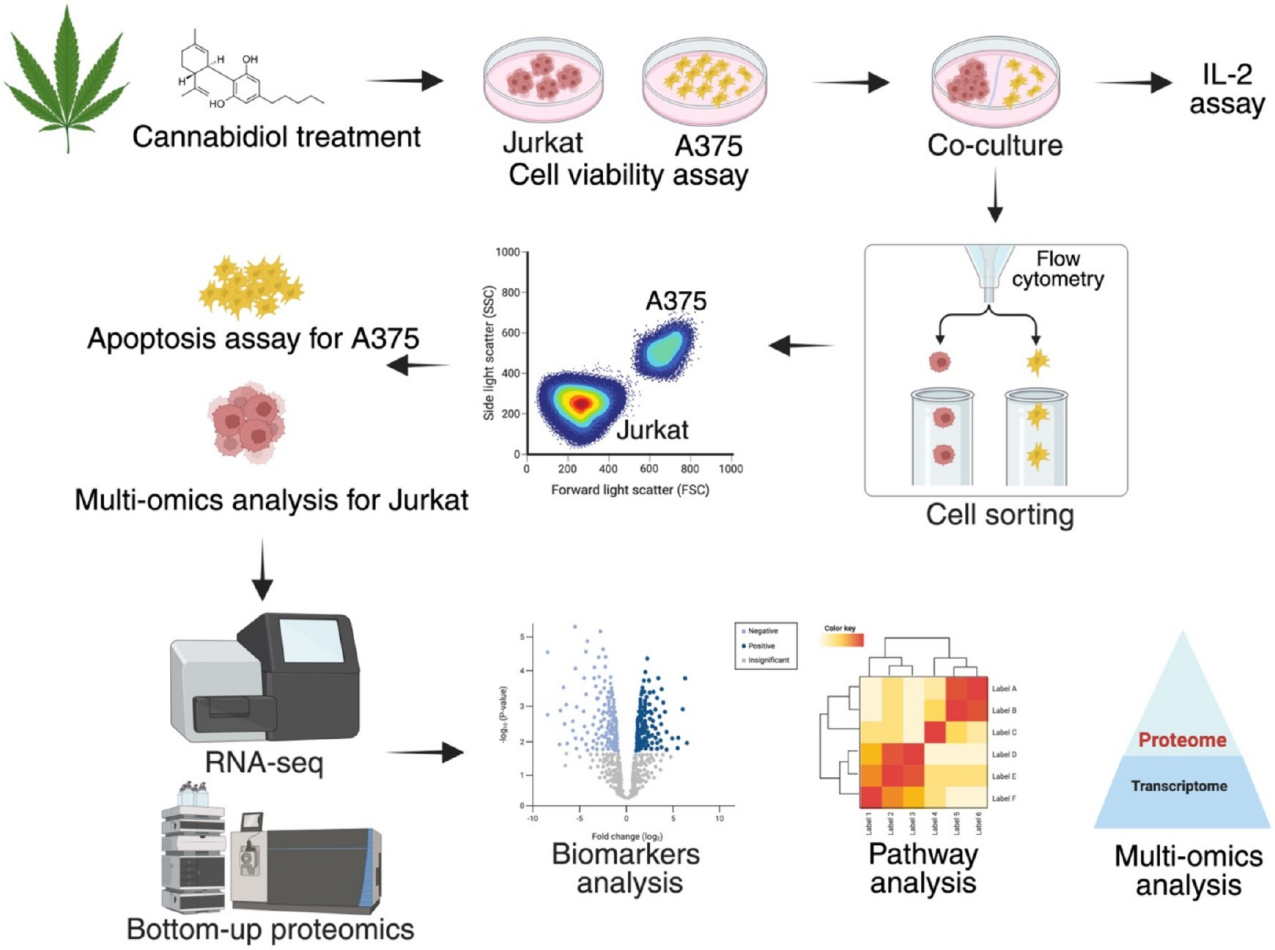
Schematic overview of the experimental workflow for evaluating the immunomodulatory effects of CBD using a multiomics approach. CBD was added to Jurkat T cells and A375 melanoma cells to assess cell viability and establish appropriate dosing conditions. Jurkat and A375 cells were then cocultured with or without CBD, followed by measurement of IL-2 secretion. Cocultured cells were separated by flow cytometry–based sorting to isolate individual cell populations (Jurkat and A375) for downstream analyses. Apoptosis assays were conducted to evaluate the cytotoxic effects of CBD. Sorted cells were subjected to transcriptomic profiling (RNA-seq) and quantitative bottom-up proteomics. Differential expression analyses, biomarker identification, pathway enrichment, and integrated multiomics analyses were performed to characterize CBD-induced molecular alterations and reveal regulatory networks associated with immune–tumor interactions.

## 2 Materials and Methods

### 2.1 Chemicals and Reagents

Cannabidiol (CBD) was purchased from Cayman Chemical (Ann Arbor, MI, USA). Anti-CD3, anti-CD28 antibodies, and ELISA Max Deluxe human IL-2 kit were purchased from BioLegend (San Diego, CA, USA). Fetal bovine serum (FBS) and Roswell Park Memorial Institute (RPMI) 1640 medium were purchased from Gibco Life Technologies (Gaithersburg, MD, USA).

### 2.2 Cell Culture and Viability Assay

Human melanoma A375 cells and human Jurkat T cells were purchased from the American Type Culture Collection (ATCC; Rockville, MD, USA) and cultured according to protocols by ATCC. The A375 cell line was cultured in DMEM medium, and the Jurkat cell line was cultured in RPMI 1640 medium. Cells were supplemented with 10% FBS at 5% CO_2_ and 37 °C as recommended by ATCC. Jurkat cell viability was evaluated using the Cell Counting Kit-8 (CCK-8; Dojindo, Rockville, MD, USA) assay. Jurkat cells were seeded in 96-well plates at a density of 1 × 10^4^ cells/well and treated with various concentrations of CBD. After a 24 h incubation, 10 μL of CCK-8 reagent was added to each well. The plate was then incubated for an additional 1–4 h at 37 °C to facilitate colorimetric development. Absorbance of each well was measured at 450 nm using a SpectraMax M2 plate reader (Molecular Devices, Sunnyvale, CA, USA) to determine cell viability.

### 2.3 Cell Coculture

A375 cells were seeded in 12-well plates at a density of 5 × 10^4^ cells/mL and allowed to adhere overnight. The following day, the cells were treated with interferon-γ (IFN-γ, 10 ng/mL) and incubated at 37 °C in a humidified CO_2_ incubator for 24 h. Jurkat cells were seeded separately in 100 mm dishes at a density of 5 × 10^5^ cells/mL and activated with anti-CD3 antibody (100 ng/mL) and anti-CD28 antibody (100 ng/mL) for 24 h. The activated Jurkat cells were then transferred to the IFN-γ-treated A375 cell cultures.

### 2.4 Cellular ROS Assessment

Intracellular ROS levels were measured using the DCFDA probe. Following coculture, cells were incubated with DCFDA (20 μM) at 37 °C for 30 min. Cellular fluorescence intensity was then measured using a SpectraMax M2 plate reader (Molecular Devices, Sunnyvale, CA, USA) with excitation and emission wavelengths of 485 and 525 nm, respectively. To assess cell-type-specific ROS levels, Jurkat T cells and A375 melanoma cells were separated after coculture and collected into individual tubes. Each cell population was incubated with DCFDA (20 μM) at 37 °C for 30 min, followed by analysis by flow cytometry using a BD FACSCalibur system (BD Biosciences, San Jose, CA, USA).

### 2.5 Cellular Immune Assays

For the measurement of IL-2, the medium containing suspended cells was collected and centrifuged at 500*g* for 3 min. The resulting supernatant was used to assess interleukin-2 (IL-2) levels using an ELISA kit (BioLegend, San Diego, CA, USA). For the detection of apoptosis, the suspension cells from the coculture were collected in a centrifuge tube. For adhered cells, phosphate-buffered saline (PBS) was used to wash twice, then trypsin without ethylenediaminetetraacetic acid (EDTA) was added to digest the cells, followed by collecting cells in a centrifuge tube by centrifuging at 500*g* for 3 min. The collected suspension and adhered cells were resuspended in annexin-binding buffer (100 μL) containing Alexa Fluor TM 488 Annexin V (5 μL) and PI working solution (1 μL). After incubation at room temperature for 15 min, the stained cells were analyzed by flow cytometry (BD Biosciences, San Jose, CA, USA).

### 2.6 T Cell Sorting

A375 cells were seeded in a 12-well plate at a density of 5 × 10^4^ cells/mL and allowed to attach overnight. IFN-γ (10 ng/mL) was added and incubated for 24 h, then cell tracker deep red (0.5 μM) was added and incubated at 37 °C for 20 min. Jurkat cells were stained with cell tracker green (1 μM) and incubated at 37 °C for 20 min. Then, cells were seeded in a 100 mm dish at a density of 5 × 10^5^ cells/mL, followed by activation with anti-CD3 antibody (100 ng/mL; 317303, BioLegend) and anti-CD28 antibody (100 ng/mL; 302913, BioLegend). Next, Jurkat cells were cocultured with A375 cells for 24 h, followed by adding CBD or DMSO for another 24 h. The suspension cells were collected in a centrifuge tube, washed with PBS 2 times for the adhered cells, then trypsin was added to digest the cells before collecting them in the centrifuge tube (centrifuge at 500*g* for 3 min). The cell pellets were resuspended in PBS (100 μL; with 5% FBS) and sorted by flow cytometry (BD Biosciences). T cells were sorted from the T cell-cancer cell coculture system using a flow cytometer in the Flow Cytometry Core (COBRE Center for Stem Cells and Aging; Brown University, Providence, RI, USA).

### 2.7 Direct Data-Independent Acquisition (DIA) Analysis

Peptides were separated on a reverse-phase Phenomenex Kinetex XB-C18 column (2.6 μm, 100 Å, 150 mm × 0.3 mm) maintained at 40 °C; the autosampler was kept at 5 °C. Separation was carried out using a 45 min linear gradient at a flow rate of 5 μL/min. Mobile phase A was water with 0.1% (v/v) formic acid, and mobile phase B was acetonitrile with 0.1% (v/v) formic acid. The gradient was programmed as follows: 0–1 min, 97% A; 1–46 min, 97 to 70% A; 46–48 min, 70 to 20% A; 48–53 min, 20% A; 54–65 min, re-equilibration at 97% A. For mass calibration, SCIEX ESI Positive Calibration Solution X500B was infused every 5 samples. Data were acquired using SCIEX OS v3.4.0. In ZenoSWATH-MS experiments, ionization was in positive mode. Source parameters were ion source gas 1, 10 psi; ion source gas 2, 20 psi; CAD gas, 7 psi; curtain gas, 35 psi; source temperature, 100 °C; spray voltage, 5000 V. MS1 survey scans were acquired from 400–1500 *m*/*z* with an accumulation time of 50 ms. DIA fragmentation was performed with 85 variable windows spanning 399.5–903.5 *m*/*z*, using a 20 ms accumulation time and dynamic collision energy.

### 2.8 DIA Analysis

Sample preparation and DIA analysis were conducted by a standard procedure, as we previously published.^25^ Chloroform/methanol protein extraction and pressure cycling technology (PCT)-aided trypsin digestion was conducted by our standard procedure. Data-independent acquisition (DIA) was performed on a SCIEX ZenoTOF 7600+ mass spectrometer equipped with an OptiFlow Turbo V ion source (SCIEX, Marlborough, MA, USA) and coupled to an ACQUITY M-Class UPLC system (Waters Corp., Milford, MA, USA). Fragment ion intensities (MS^2^ signals) were extracted from the DIA data using Pulsar with default settings. The resulting report, exported in. tsc format, was used as input for the msDiaLogue package (https://github.com/uconn-scs/msDiaLogue), developed by the Center for Open Research Resources & Equipment at the University of Connecticut. The exported data included the following fields: R.Condition, R.Replicate, PG.Genes, PG.ProteinAccessions, PG.ProteinDescriptions, PG.ProteinNames, PG.NrOfStrippedSequencesIdentified, and PG.Quantity. Within the msDiaLogue pipeline, a stringent filtering criterion was applied to retain only proteins identified by at least two stripped peptide sequences, enhancing the reliability of protein identification. The pipeline also included protein-level filtering, normalization, and statistical testing to identify proteins with differential abundance between CBD-treated and control groups. Data visualization was carried out using volcano plots and heatmaps of the top-expressed proteins. An unpaired *t*-test was used to compare the two groups, with significance thresholds set at a fold-change of ≥ 1.5 (log_2_ ≥ 0.58) and a *p*-value <0.05.

### 2.9 RNA-seq Library Preparation and Sequencing

RNA-seq libraries were prepared using KAPA/Roche mRNA HyperPrep Kit (KAPA/Roche, 8098123702) according to the manufacturer’s instructions. Briefly, 1 μg of total RNA was purified using magnetic oligo-dT beads. Purified mRNA was then fragmented at 94 °C for 6 min, after which it was placed immediately on a magnet, and the supernatant was transferred to a new tube on ice. First strand synthesis was performed, followed by a second strand synthesis. Diluted KAPA unique dual indexes (7 μM; KAPA/Roche, 8861919702) were added via ligation, and the product was purified using KAPA Pure Beads (KAPA/Roche, 7983298001). The purified product was then amplified, bead-purified, and finally, eluted in Tris-Cl (10 mM, 20 μL, pH 8.5; Qiagen, 19086). Libraries were then quantified using a Qubit v2.0 (ThermoFisher) with a dsDNA HS assay kit (ThermoFisher, Q32854) and validated using a TapeStation 4200 (Agilent, G2991Ba, Santa Clara, CA, USA) with a High Sensitivity D5000 ScreenTape and associated reagents (Agilent, 5067–5592­(3,4)). For RNA-seq, samples were sequenced at the Hubbard Center for Genome Studies (University of New Hampshire, Durham, NH, USA) on a NovaSeq 6000 (Illumina; San Diego, CA, USA) using paired-end, version 1.5 chemistry on an SP, patterned flow cell. Forward and reverse read lengths were 250 base pairs, and indexing reads were dual 8-mers. The data were demultiplexed using Illumina bcl2fastq v2.20.0.422.

### 2.10 RNA-seq Data Analysis

Raw sequencing data were processed using a reproducible Snakemake workflow. Reads were first trimmed for adapters and low-quality bases using Trim Galore, followed by quality control checks with FastQC. Clean reads were aligned to the human reference genome (GRCh38) using HISAT2. SAM files were sorted and converted to BAM format using SAMtools, and transcript assembly and quantification were performed with StringTie. Gene-level count matrices were generated using the prepDE.py script. Downstream analysis was conducted in R using DESeq2. Genes were filtered and normalized, and differential expression was computed using shrunken (via the apeglm method) log_2_ fold change estimates. Principal component analysis (PCA) and heatmaps with the top significantly expressed genes were generated using variance-stabilizing transformed data. A volcano plot was generated to show significantly differentially expressed genes, defined by an adjusted *p*-value <0.05 and an absolute log_2_ fold change >0.58.

### 2.11 Multiomics Integrated Pathway Analysis

Both proteomics and transcriptomics data sets were uploaded into Ingenuity Pathway Analysis (IPA) for core analysis. The proteomics data set included 4366 mapped protein accession IDs with associated fold changes (treatment vs control) and *p*-values. The transcriptomics data set included 17,691 mapped gene symbols, also with corresponding fold changes and *p*-values. To obtain a comprehensive view of CBD’s effects on T cells, no filtering was applied to genes or proteins in these analyses. A comparison analysis was then performed to integrate the results from the proteomics and transcriptomics core analyses. This allowed for direct comparison of canonical pathways, upstream regulators, molecules, and networks identified in both data sets.

### 2.12 Statistical Analysis

Data are shown as mean ± standard deviation (S.D). A *p*-value of less than 0.05 was considered statistically significant between the two groups.

## 3 Results

### 3.1 Cannabidiol Exhibits Concentration-Dependent Cytotoxic and Cytoprotective Effects in Melanoma and T Cells

To define pharmacologically relevant concentrations for coculture experiments, we first assessed CBD cytotoxicity in A375 melanoma cells and Jurkat T cells. Consistent with reported CBD cytotoxicity,^26^ CBD reduced Jurkat viability in a concentration-dependent manner, with an IC_50_ of 20.2 μM (Figure 2A). At concentrations ≥ 12.5 μM, Jurkat viability declined markedly, whereas concentrations ≤ 10 μM produced minimal cytotoxicity and even slightly increased viability relative to control. Based on these findings, 10 μM CBD was selected as a functionally noncytotoxic concentration for subsequent coculture and multiomics analyses.

**Figure 2:**
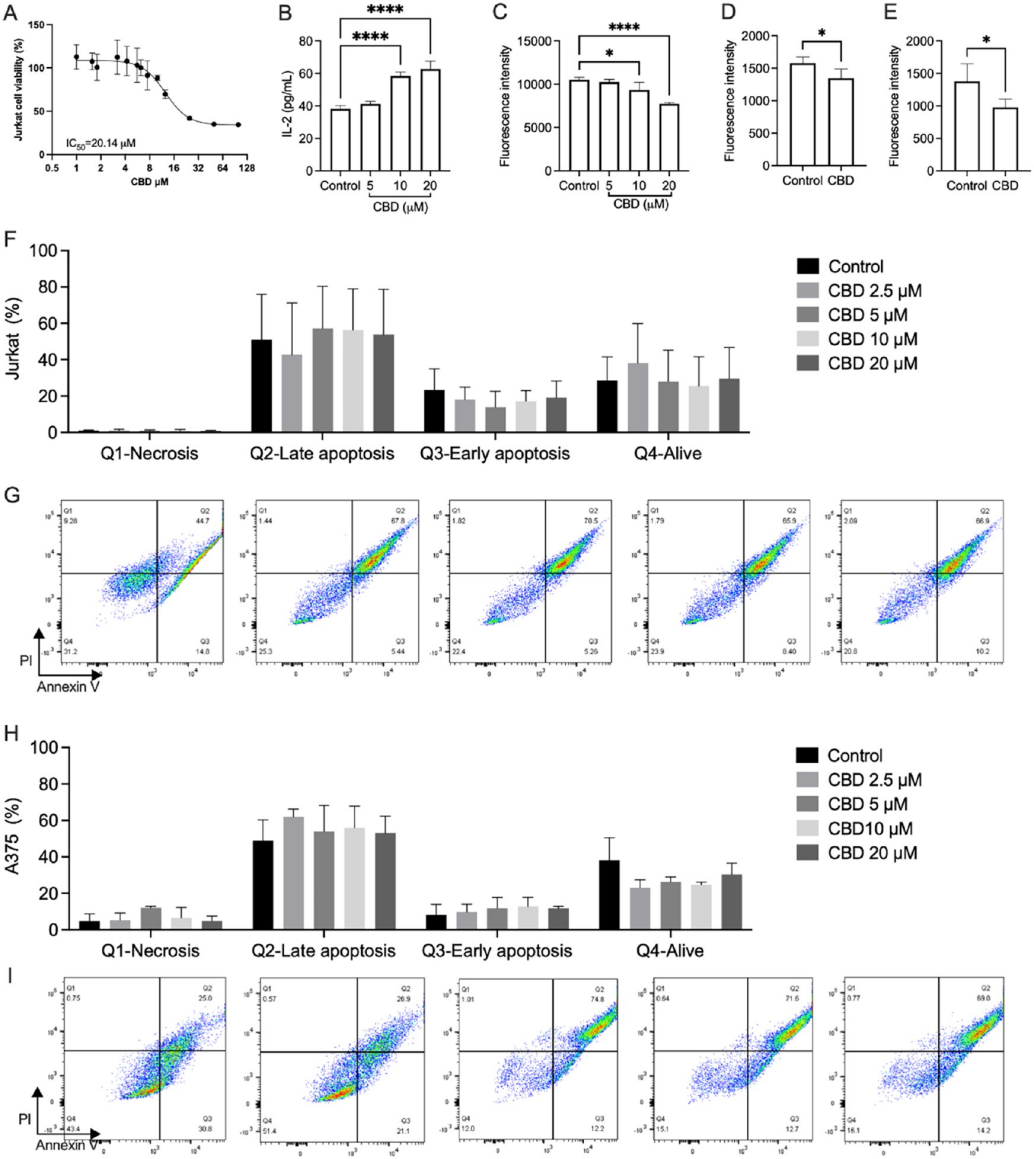
Effects of CBD on Jurkat T-cell viability, cytokine production, and apoptosis in Jurkat and A375 cells. (A) Concentration–response curve of Jurkat cell viability following CBD treatment (0.5–128 μM) for 24 h, showing an IC_50_ of 20.2 μM. (B) IL-2 secretion from Jurkat cells treated with increasing concentrations of CBD (5–20 μM). CBD at 10 and 20 μM enhanced IL-2 production (**** *p* < 0.0001), whereas 5 μM showed no significant difference from control. (C) Effects of CBD on the total production of ROS in the coculture system. (D) Quantitative analysis of ROS from A375 cells detected by fluorescence signals. Data are shown as the averaged FITC values. (E) Quantitative analysis of ROS from Jurkat cells detected by FITC fluorescence values. (F) Quantification of apoptosis in Jurkat cells treated with CBD, assessed by AV and PI staining. Quadrants represent necrosis (Q1), late apoptosis (Q2), early apoptosis (Q3), and live cells (Q4). (G) Flow cytometry dot plots of apoptosis in Jurkat cells treated with CBD. (H) Quantification of apoptosis in A375 melanoma cells treated with CBD. (I) Flow cytometry dot plots of apoptosis in A375 melanoma cells treated with CBD.

### 3.2 Cannabidiol Modulates Cytokine Secretion in a Melanoma–Immune Coculture System

To investigate the effect of CBD on immunoregulation, we measured IL-2 levels in the supernatant of the coculture model. Given that cell viability ranged from 117 to 34.4% across CBD concentrations from 1 to 100 μM (Figure 2A), we selected a narrower range (5, 10, and 20 μM) to evaluate its concentration-dependent effects. CBD treatment resulted in IL-2 levels of 41.1, 58.4, and 62.7 pg/mL, respectively, compared to 38.1 pg/mL in the untreated group (Figure 2B), suggesting that CBD modulated immune response in the coculture model in a concentration-dependent manner.

### 3.3 Cannabidiol Decreases ROS Expression in a Melanoma–Immune Coculture System

To evaluate the effect of CBD on oxidative stress, intracellular ROS levels were measured in the coculture model. CBD treatment at 5, 10, and 20 μM resulted in concentration-dependent reductions in ROS levels by 2.7, 11.2, and 26.5%, respectively (Figure 2C). To further delineate cell-type-specific responses, ROS levels were assessed separately in A375 melanoma cells and Jurkat T cells by flow cytometry. In A375 cells, CBD (10 μM) reduced ROS levels by 14.6% compared to the vehicle control (Figure 2D). In Jurkat T cells, a greater reduction of 26.8% was observed at the same concentration (Figure 2E), suggesting enhanced sensitivity of T cells to CBD-mediated modulation of oxidative stress.

### 3.4 Cannabidiol Selectively Promotes Stress-Associated Cell Death in Melanoma Cells

To determine how CBD impacts cell death in each population, Jurkat and A375 cells were sorted from the coculture and analyzed by Annexin V/PI staining (Figure 2F–I). In Jurkat cells, CBD at 2.5–10 μM modestly decreased early and late apoptotic fractions and increased the proportion of viable cells, with more pronounced toxicity only at 20 μM (Figure 2F–G). In contrast, A375 melanoma cells exhibited a different response. CBD induced a concentration-dependent increase in necrosis and apoptosis, particularly at 5–10 μM, accompanied by a marked reduction in viable A375 cells (Figure 2H–I). Importantly, these findings define a pharmacological window in which CBD preferentially induces tumor cell death while preserving and functionally activating T cells. At 10 μM, CBD promoted melanoma cytotoxicity while maintaining T-cell viability and enhancing IL-2 secretion, suggesting differential stress tolerance between tumor and immune compartments within the coculture system.

### 3.5 Cannabidiol Induces Stress-Adaptive Transcriptomic Remodeling in T Cells

Jurkat T cells isolated from the melanoma-T cell coculture were subjected to RNA-seq to characterize CBD-induced transcriptional changes. PCA demonstrated partial separation between CBD-treated and control T cells (PC1 = 40%, PC2 = 16%; Figure 3A), indicating global transcriptomic remodeling. Differential expression analysis identified 220 DEGs (182 upregulated, 38 downregulated) in CBD-treated T cells (adjusted *p* < 0.05, |log_2_FC| > 0.58; Figure 3B). Visualization of the top differentially expressed genes revealed coordinated transcriptional changes consistent with stress-adaptive T-cell activation and remodeling of migratory programs (Figure 3C). CBD downregulated genes linked to T-cell homing and signaling restraint, including CCR9, DTX1, HES4, and RASAL1, while inducing genes involved in metabolic adaptation, cellular stress responses, and effector function. This suggests that CBD elicited transcriptional reprogramming of T cells toward a more activated and migratory phenotype under tumor-associated stress conditions.

**Figure 3:**
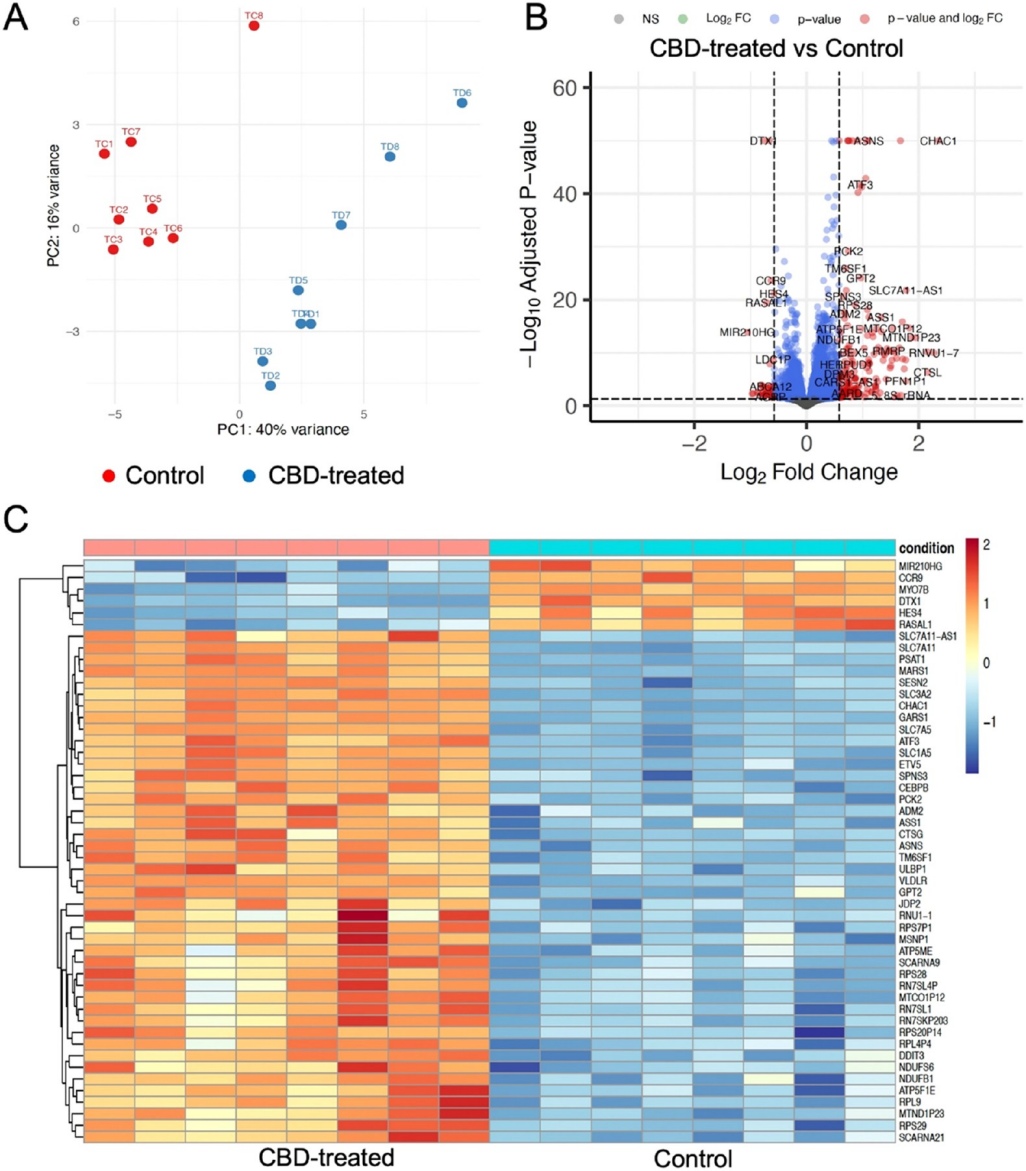
Transcriptomic profiling reveals distinct gene expression signatures in Jurkat T cells following CBD (10 μM) treatment. (A) PCA of RNA-seq data showing partial separation between control and CBD-treated Jurkat T cells. Biological replicates cluster tightly within each condition, indicating high data set reproducibility. (B) Volcano plot illustrating differentially expressed genes (DEGs) in CBD-treated cells compared with controls. Red points represent significantly upregulated genes and blue points represent significantly downregulated genes (adjusted *p*-value <0.05 and |log_2_ fold change| ≥1). Several CBD-responsive genes implicated in immune regulation, stress signaling, and metabolic pathways are highlighted. (C) Heatmap of the top DEGs (ranked by adjusted *p*-value) demonstrating distinct expression patterns between groups. Hierarchical clustering shows coordinated upregulation and downregulation of gene modules in CBD-treated cells.

### 3.6 Transcriptomic Analysis Links Cannabidiol Exposure to Immune Signaling and Cellular Trafficking Pathways

To gain mechanistic insight into CBD-mediated transcriptional changes, we performed pathway and network analysis of the RNA-seq data. IPA revealed broad modulation of signaling nodes across cellular compartments. CBD upregulated multiple cytokines (CSF2, IL4, TNF, VEGFA, KITLG), the costimulatory receptor CD28, and nuclear transcriptional regulators such as JUN, TP53, MYC, and ATF4, while repressing epigenetic and transcriptional regulators including DNMT3A, HELLS, and FOXO4 (Figure 4A). Canonical pathway analysis highlighted several pathways directly relevant to T-cell function and tumor immunity, including T-cell receptor (TCR) signaling, noncanonical NF-κB signaling, TNFR2 noncanonical NF-κB signaling, class I MHC–mediated antigen processing and presentation, granzyme A signaling, and RUNX1-dependent transcription (Figure 4B). These findings indicate that CBD enhances core T-cell activation and effector programs within the melanoma coculture system. Machine learning (ML)–based disease and function analysis further identified immunosuppression as a significantly enriched functional category in CBD-treated cells (Figure 4C,D). Key mediators predicted to contribute to this signature included IL-10, PPP3CA/B/C, and ADORA2A, alongside stress-response and translational regulators such as EIF2A, EIF2B1, and EIF2S1, suggesting engagement of counter-regulatory and stress-adaptive mechanisms.

**Figure 4:**
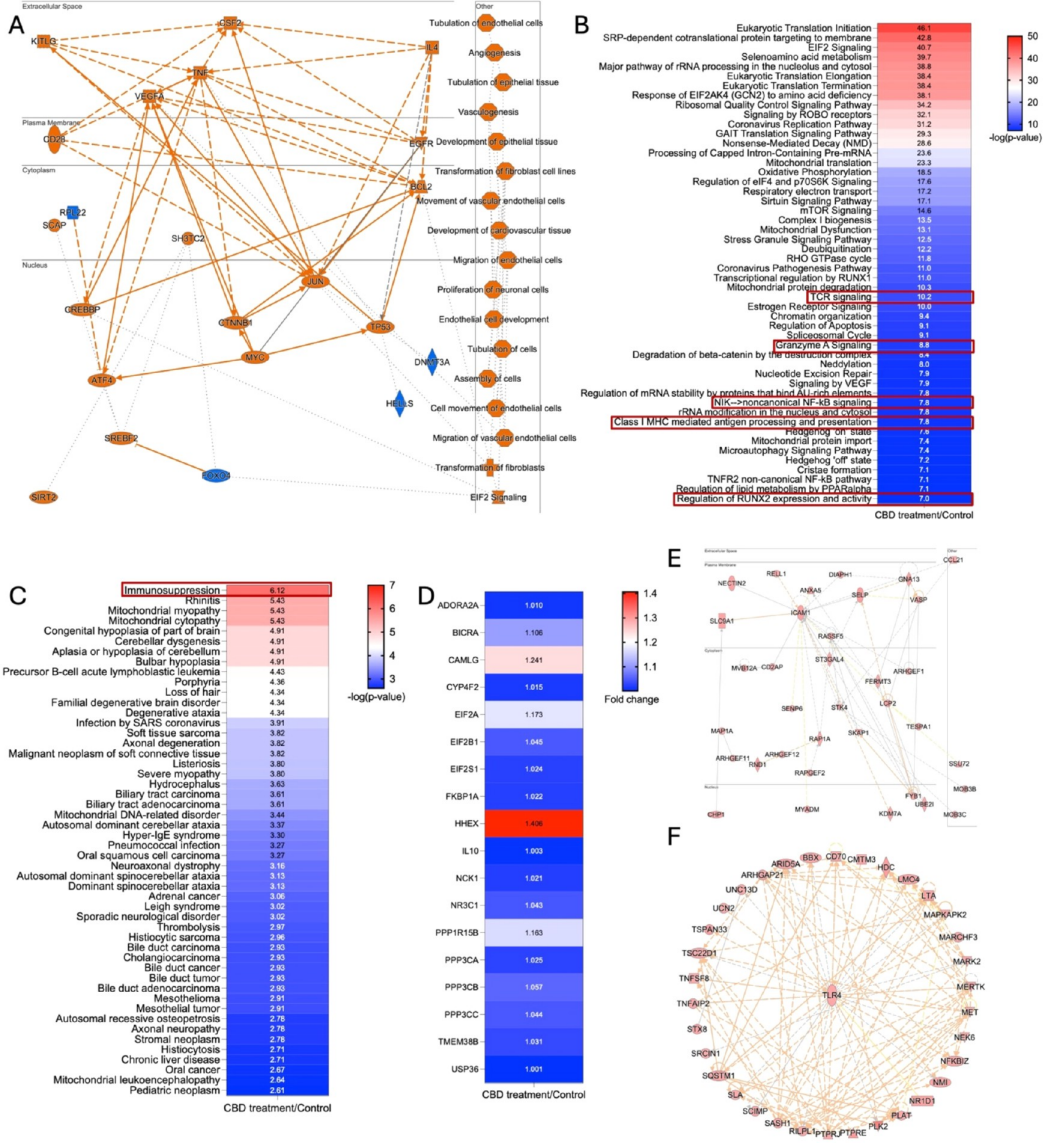
Integrated pathway and upstream regulator analysis identifies CBD-responsive signaling networks in Jurkat T cells. (A) IPA of differentially expressed genes revealed activation of signaling networks. Nodes are colored by predicted activation state (orange: activated; blue: inhibited), and edge thickness reflects interaction confidence. (B) Top enriched canonical pathways ranked by – log_10_(*p*-value). (C) IPA disease and biological function enrichment analysis showing predicted functional associations affected by CBD treatment. (D) Heatmap of top predicted upstream regulators and key transcriptional drivers influenced by CBD, displaying fold-change directionality. Regulators include cytokines, metabolic enzymes, translation, and stress-response mediators. (E) IPA regulatory network centered on EIF2 signaling, illustrating CBD-dependent modulation of translation control, stress adaptation, and antigen presentation machinery. (F) Expanded interaction network of differentially expressed genes depicting broader molecular connectivity and signal integration across immune, metabolic, and stress-response pathways.

Network analysis of the RNA-seq data identified 25 networks (see details in Supporting Information Table S1), with two (networks 10 and 13) enriched for immune cell trafficking (Figure 4E,F). CBD upregulated CCL21 and ICAM1 in network 10, both critical drivers of immune cell migration and adhesion. CCL21 establishes chemokine gradients that guide T-cell homing, whereas ICAM1 mediates firm adhesion and transendothelial migration through interactions with integrins. In network 13, activation of TLR4 and MERTK, regulators of innate sensing and apoptotic cell clearance, further implicated CBD in reshaping the migratory and surveillance behavior of immune cells within the melanoma coculture. Collectively, these transcriptomic data indicate that CBD reprograms T cells toward enhanced activation and altered trafficking, while simultaneously engaging immunosuppressive and stress-response pathways.

### 3.7 Proteomics Reveals CBD-Mediated Regulation of EIF2 Signaling and Immune Trafficking Nodes

To complement transcriptomic findings, we performed DIA-based bottom-up proteomics on T cells isolated from the coculture. PCA again demonstrated partial separation and distinct clustering trends between control and CBD-treated samples along PC1 (36%) and PC2 (16%) (Figure 5A). Differential expression analysis identified 25 differentially expressed proteins (14 upregulated, 11 downregulated; Figure 5B), with CBD-induced changes generally more modest at the proteome level than at the transcriptome level. A heatmap of the top 50 differentially expressed proteins (Figure 5C) confirmed distinct CBD-driven proteomic signatures, indicating that CBD elicits coherent but attenuated protein-level responses relative to mRNA.

**Figure 5:**
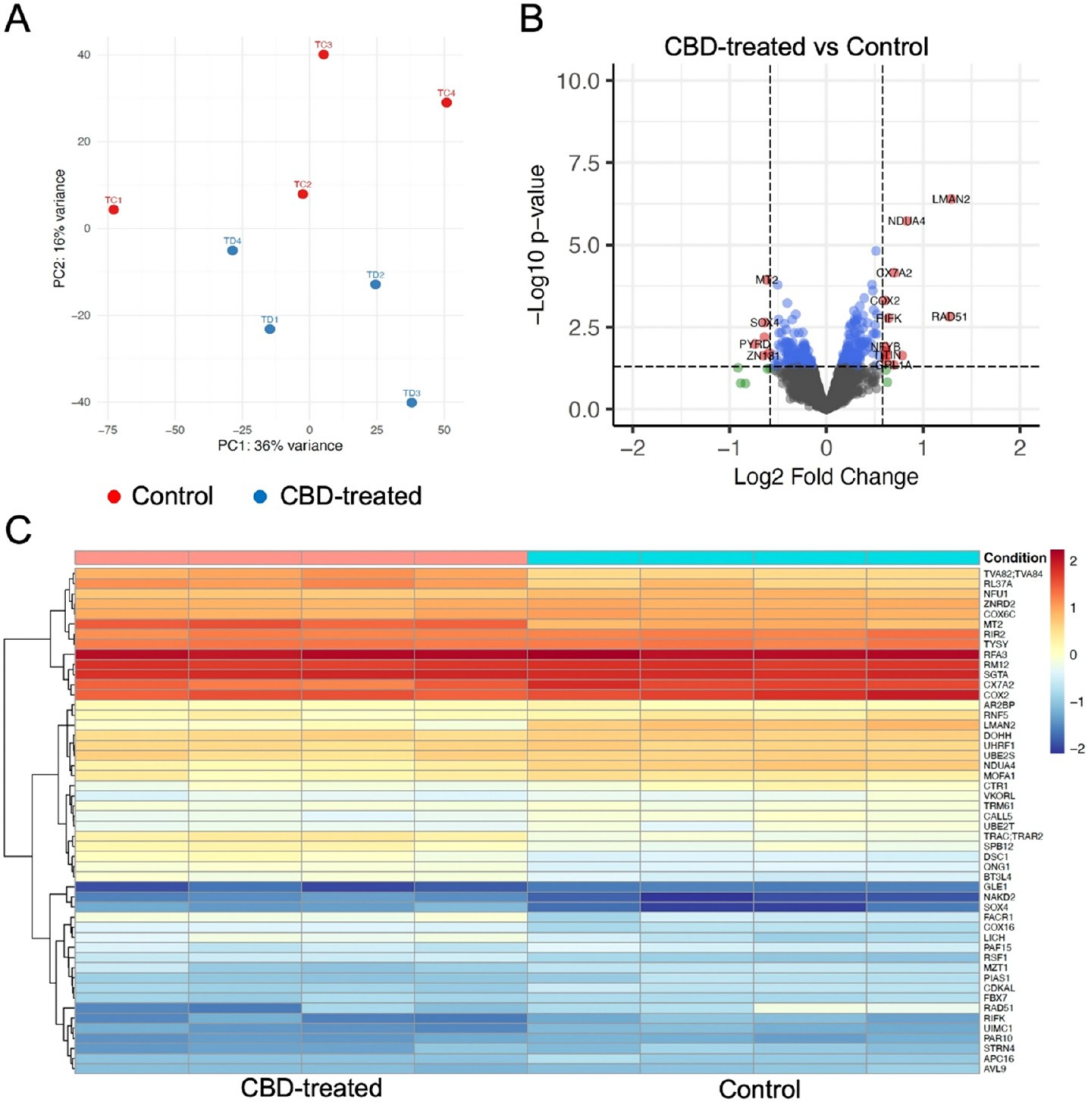
Proteomic profiling reveals distinct CBD-induced molecular signatures in Jurkat T cells. (A) PCA of quantitative proteomics data showing partial separation between control and CBD-treated Jurkat T cells. (B) Volcano plot displaying DEPs in CBD-treated cells relative to control. Proteins meeting significance thresholds (*p* < 0.05 and |log_2_ fold change| ≥1) are highlighted, with several CBD-responsive proteins annotated, including regulators of mitochondrial function, immune signaling, and stress response pathways. (C) Heatmap of top DEPs ranked by adjusted *p*-value, illustrating CBD-driven changes in protein abundance.

IPA of the proteomics data set revealed prominent modulation of pathways controlling cell cycle progression and stress-responsive translation (Figure 6). CBD treatment was associated with downregulation of E2F1 and related cell-cycle regulators, suggesting attenuation of S-phase progression, and with activation of EIF2 signaling. This activation indicates engagement of PERK–eIF2α–ATF4–related translational control, a canonical arm of the ISR that coordinates adaptive protein synthesis under oxidative and ER stress conditions. In T cells, this axis regulates activation thresholds, metabolic adaptation, and cytokine production, suggesting that CBD-driven ISR engagement may underlie the observed functional remodeling. Within the EIF2 pathway, a substantial fraction of detected components showed altered abundance, and markers of ER stress were upregulated, consistent with CBD-induced translational reprogramming under stress conditions.

**Figure 6:**
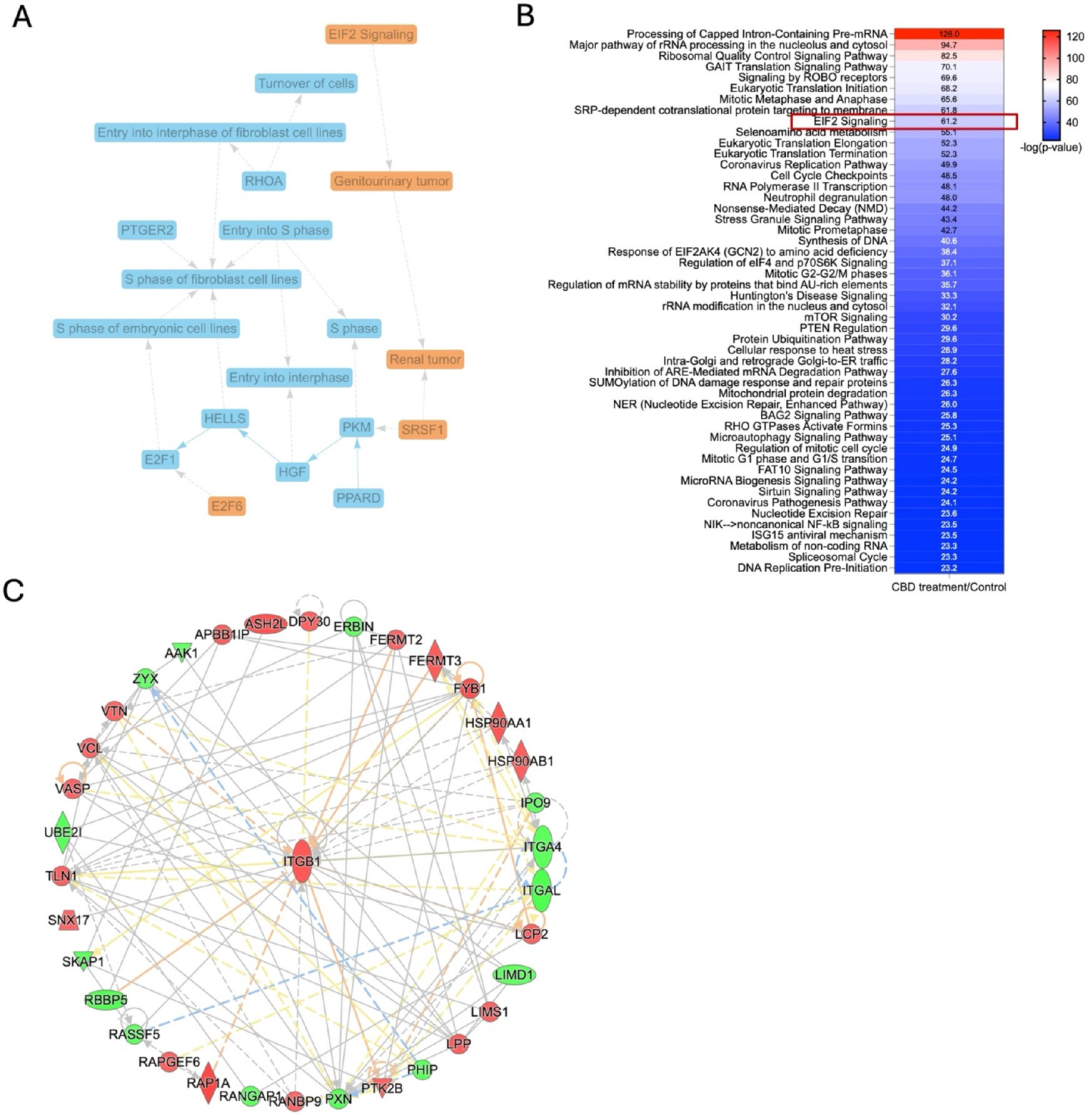
Pathway enrichment and network modeling identify CBD-responsive signaling cascades at the proteome level. (A) IPA upstream regulator and functional network model based on differentially expressed proteins in CBD-treated Jurkat T cells. The network highlights predicted activation (orange) or inhibition (blue) of key regulators involved in cell cycle progression, S-phase entry, genitourinary tumor signaling, and EIF2-dependent stress responses. Node color reflects predicted activation state, and edges represent curated molecular interactions. (B) Top enriched canonical pathways ranked by – log_10_(*p*-value). (C) Comprehensive IPA protein interaction network illustrating integration of CBD-responsive regulators across subcellular compartments. Nodes are colored by predicted activation state (red: activated; green: inhibited), and edge coloring indicates activating (orange), inhibitory (blue), or undirected (gray) interactions.

Network analysis identified 25 proteomic networks, among which network 19 was enriched for immune cell trafficking–related molecules and centered on ITGB1 (Figure 6C). ITGB1 is essential for leukocyte adhesion, migration, and extravasation. Additional key components included talin-1 (TLN1), which activates integrins by binding their cytoplasmic tails, and FYB1, an adaptor that couples TCR and chemokine receptor signaling to integrin activation. These proteomic findings, together with the transcriptomic data, support a model in which CBD modulates adhesion and trafficking machinery in T cells, potentially enhancing their ability to interact with and infiltrate tumor tissue.

### 3.8 Integrated Multiomics Analysis Reveals Convergent Stress-Adaptive and Trafficking Responses to Cannabidiol

To integrate transcriptomic and proteomic responses to CBD, we compared canonical pathways, upstream regulators, and networks across both data sets (see Supporting Information Tables S2, S3, S4, and S5). More than 600 canonical pathways were detected, and many showed stronger regulation at the transcript level than at the protein level. For example, neutrophil degranulation and TCR signaling exhibited high activation scores in RNA-seq data but only modest changes in the proteome (Figure 7A), demonstrating classic transcript-protein divergence driven by post-transcriptional and translational control. Upstream regulator analysis revealed a set of transcription factors and coregulators (e.g., MYC, MLXIPL, SPEN, MYCL, SRSF1, CREM) predicted to be activated in CBD-treated T cells at the transcriptomic level, with several also moderately supported by proteomic data (Figure 7C). These regulators are intimately linked to T-cell activation, metabolism, and RNA processing, aligning with the broader pathway changes observed.

**Figure 7:**
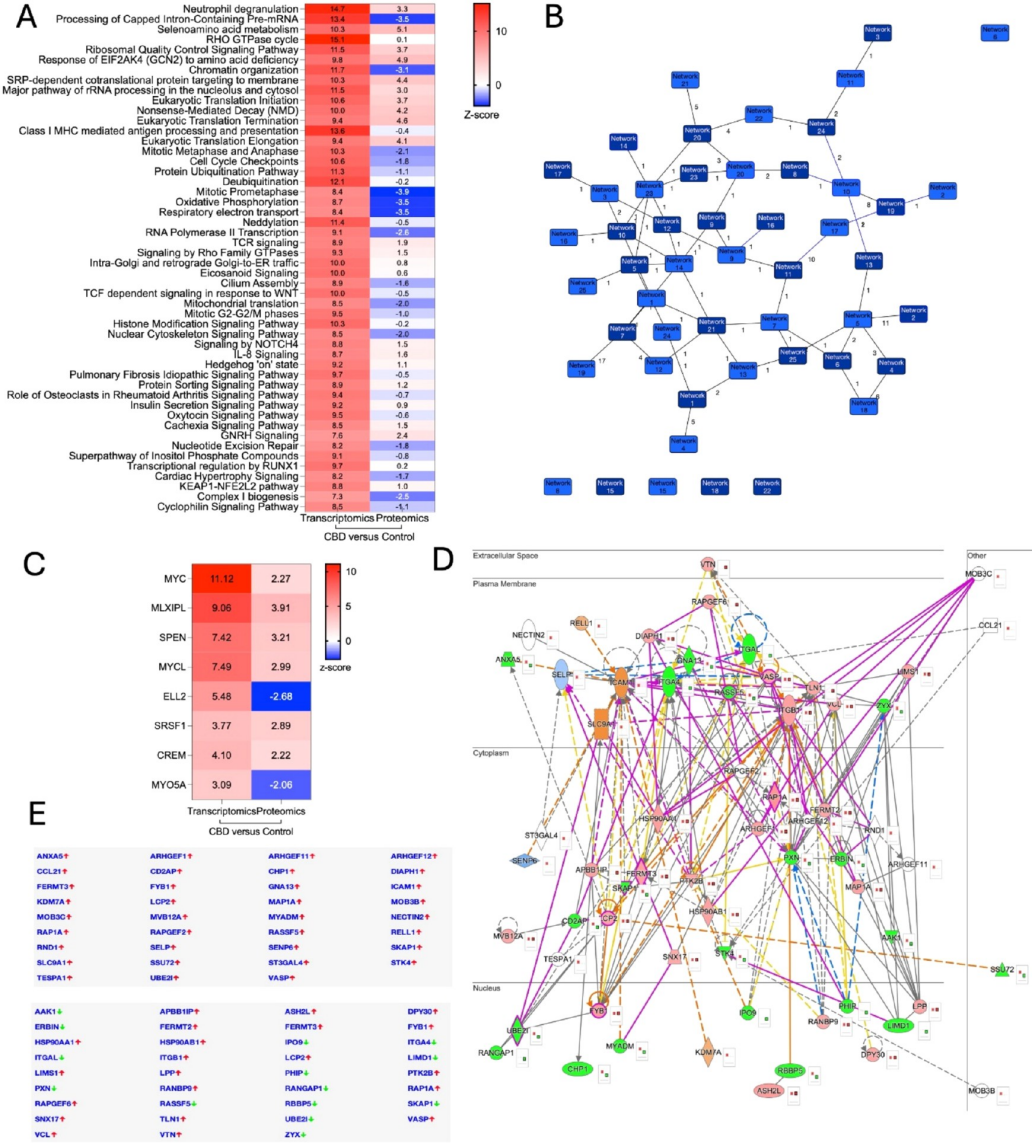
Integrated transcriptomic–proteomic analysis identifies convergent CBD-regulated pathways and upstream regulatory networks. (A) Heatmap of top canonical pathways identified by integrating transcriptomic and proteomic data sets, ranked by activation Z-score. Pathways related to immune activation, antigen processing, and stress-responsive signaling also showed coordinated alterations across both omics layers. (B) Network map of coregulated canonical pathways illustrating the interconnectivity of CBD-responsive biological processes. Nodes represent pathways, and edges indicate functional overlap based on shared gene or protein members. (C) Predicted upstream regulators identified through integrated multiomics analysis, displayed with Z-scores from transcriptomic (left) and proteomic (right) data sets. (D) IPA regulatory network highlighting CBD-modulated interactions involving immune checkpoints, adhesion molecules, stress-related transcription factors, and metabolic enzymes. Nodes are colored by predicted activation state (orange: activated; green: inhibited), and arrows indicate directionality of regulatory influence. (E) Summary of significantly altered gene–protein pairs showing concordant directional changes across both data sets.

Notably, integrated network analysis identified immune cell trafficking as a convergent theme across transcriptomic and proteomic data sets. Merging transcriptomic network 10 with proteomic network 19 revealed coordinated upregulation of ICAM1 and ITGB1 as central nodes linking stress signaling to adhesion and migratory machinery (Figure 7E). Both molecules are redox-sensitive regulators of leukocyte adhesion and extravasation, providing a functional bridge between ISR engagement and immune cell positioning. The concordant regulation of FERMT3, FYB1, LCP2, RAP1, and VASP further supports coordinated remodeling of integrin activation and cytoskeletal dynamics. Together, these findings suggest that CBD-induced stress signaling translates into functional modulation of immune trafficking pathways relevant to tumor–immune engagement. Overall, the integrated multiomics data suggest that CBD reprograms T-cell signaling at both transcriptomic and proteomic levels, with convergent enhancement of TCR-associated signaling and immune trafficking modules. The consistent induction of ICAM1 and ITGB1 suggests that CBD may promote T-cell–tumor engagement and trafficking, features that are directly relevant to the optimization of melanoma immunotherapy.

## 4 Discussion

In this study, we present a systems-level analysis of how CBD reshapes T-cell function within the melanoma microenvironment through redox- and stress-responsive mechanisms. Using a melanoma-T cell coculture model integrated with transcriptomic and proteomic analyses, we demonstrate that CBD selectively promotes melanoma cell death while inducing coordinated stress-adaptive signaling programs in T cells. Rather than acting solely through direct cytotoxic effects, CBD reprograms immune cell signaling, translation, and trafficking networks, revealing a multifaceted mode of action rooted in redox biology.

Consistent with our previous reports, CBD exhibited moderate cytotoxicity toward A375 melanoma cells,^26^ while T-cell viability was preserved at subcytotoxic concentrations. Interestingly, moderate effects were also observed in Jurkat cells, in agreement with earlier findings that immune cell viability (monocytes and lymphocytes) is affected only at higher CBD concentrations.^26^ Within the coculture system, CBD enhanced melanoma cell apoptosis and necrosis while simultaneously increasing IL-2 secretion by T cells, showing altered immune functional output under tumor-associated stress conditions. Our findings corroborate earlier observations that cannabinoids induce melanoma cell apoptosis^27^ and extend them by demonstrating parallel immunomodulatory effects on T cells.^28^ Moreover, prior work has demonstrated a concentration-dependent reduction in melanoma cell viability mediated through CB1, TRPV1, and PPARα receptors. Our findings extend this body of work by demonstrating that CBD also modulates T-cell activity in the presence of tumor cells, suggesting that its biological effects encompass both tumor-intrinsic and immune-mediated components.

To obtain a comprehensive overview of mRNA expression changes, T cells were isolated from the coculture system and subjected to RNA sequencing. CBD treatment induced significant global alterations in gene expression. Among the most significantly altered genes, CBD downregulated regulators of homing and signaling restraint (e.g., *CCR9, DTX1, HES4*) while upregulating metabolic and stress-response genes such as *SLC7A11* and *PSAT1*. Pathway analysis using IPA further revealed broad transcriptomic changes across cellular compartments, including upregulation of cytokines (*CSF2, IL-4, TNF, VEGFA, KITLG*), membrane receptors (*CD28, EGFR*), cytoplasmic regulators (*BCL2, SCAP*), and nuclear oncogenes and transcription factors (*JUN, TP53, MYC*), while suppressing *DNMT3A*, *HELLS*, and *FOXO4*. Comprehensive pathway and network analyses highlighted significant enrichment of T cell–related signaling pathways (TCR signaling, NF-κB, antigen processing, granzyme A signaling, RUNX1 regulation), activation of immunosuppressive regulators (*IL10, ADORA2A, PPP3CAs*), and upregulation of immune trafficking molecules (*CCL21, ICAM1, TLR4, MERTK*), underscoring CBD’s dual role in both immune suppression and enhancement of immune cell trafficking within the melanoma microenvironment. These findings likely reflect a coordinated dual-modulatory state in which effector activation and regulatory counterbalancing are coinduced within a coupled feedback framework. As a redox-active and stress-inducing agent, CBD may simultaneously promote early effector programs through NF-κB and TCR-dependent signaling while engaging homeostatic regulatory pathways, including adenosine signaling (ADORA2A) and IL-10, to constrain excessive inflammatory responses.^29,30^ This interpretation is consistent with the observed increase in IL-2 secretion in the coculture system, suggesting that effector output is functionally preserved despite concurrent regulatory pathway engagement. Future studies employing single-cell RNA sequencing would help determine whether activation and suppression signatures segregate across distinct T-cell subpopulations.

Previous studies have primarily focused on CBD- or tetrahydrocannabinol (THC)-induced mRNA changes in melanoma cells.^27,31^ For example, previous transcriptomic studies of CBD or THC in melanoma cells similarly reported activation of ER stress and apoptotic pathways.^27^ In addition, treatment with a combination of THC and CBD inhibited phosphorylation of the ERK1/2 signaling pathway, which is critical for melanoma cell proliferation.^27^ Another RNA-seq analysis demonstrated that CBD induced ER stress responses, suggesting a potential redox mechanism for CBD-mediated apoptosis in skin cancers.^31^ Our results extend these findings by showing that CBD’s effects are not limited to direct action on melanoma cells but also involve modulation of T-cell immune function, thereby expanding its potential as an adjunctive immunomodulator.

Proteomic analysis identified modulation of EIF2 signaling, a central node of the ISR, linking oxidative and endoplasmic reticulum stress to translational control.^32,33^ These findings align with prior studies reporting CBD-mediated regulation of EIF2 in neurological damage^34^ and cannabidiolic acid–mediated EIF2 regulation in cancer models,^35^ supporting a conserved role for this axis in CBD’s biological activity. Integration of transcriptomic and proteomic data sets identified immune cell trafficking as a mechanistic convergence point. Adhesion and cytoskeletal regulators (i.e., ICAM1, ITGB1, FERMT3, FYB1, LCP2, RAP1, and VASP) were consistently upregulated across molecular layers. ICAM1 and ITGB1 are redox-sensitive regulators of leukocyte adhesion and extravasation, providing a functional bridge between ISR engagement and immune cell positioning. The coordinated induction of these molecules suggests that CBD-driven stress signaling extends beyond intracellular adaptation to modulate T-cell migratory capacity and tumor engagement. Direct ROS measurements using DCFDA in the coculture system demonstrated a significant reduction in intracellular ROS levels following CBD treatment. Also, the combined activation of EIF2/ATF4 signaling, ER stress pathways, and adhesion machinery supports a redox-dependent adaptive stress response.

Compared with the transcriptome, proteomic responses were attenuated. This is consistent with divergence between transcriptomic and proteomic responses, a phenomenon commonly observed in multiomics studies, where changes in mRNA and protein expression may align or diverge.^36–38^ Differences in coverage depth and post-transcriptional regulation likely contributed to this attenuation. RNA sequencing captured >17,000 transcripts, whereas proteomics quantified ∼4300 proteins, limiting direct overlap. In addition, RNA and protein were derived from parallel rather than identical biological samples, which may have increased variability. Importantly, this divergence may itself reflect ISR-mediated translational control, reinforcing the central role of stress-adaptive post-transcriptional regulation in shaping T-cell responses to CBD. The attenuated proteomic response relative to the transcriptome may reflect active post-transcriptional regulation. CBD-induced stress signaling, particularly through the ISR and eIF2α phosphorylation, is known to suppress global cap-dependent translation while selectively promoting the translation of stress-responsive mRNAs containing upstream open reading frames, such as ATF4.^39–41^ In addition, post-transcriptional mechanisms, e.g., regulation of mRNA stability by RNA-binding proteins, microRNA-mediated silencing, and alternative splicing, may further contribute to the observed divergence between transcriptomic and proteomic profiles.^42^ Future studies employing ribosome profiling (Ribo-seq) or polysome fractionation coupled with sequencing would enable direct, genome-wide assessment of translation efficiency, facilitating the identification of CBD-responsive transcripts that are actively translated versus transcriptionally buffered. Such approaches would further strengthen the mechanistic interpretation of the multiomics landscape. Collectively, our findings support a model in which CBD functions as a redox-active pharmacological modulator that engages adaptive ISR-dependent transcriptional and translational networks to reshape T-cell signaling and trafficking. The resulting phenotype reflects coordinated activation of effector, regulatory, and migratory programs under controlled stress conditions. From a translational perspective, selective engagement of adaptive stress signaling while preserving T-cell viability suggests that CBD may modulate tumor–immune interactions without broadly suppressing immune function. These properties warrant further investigation using in vivo melanoma models, particularly in the context of immune checkpoint therapy.

This study has several limitations. First, the number of proteomics samples was limited to four, given that it was challenging to sort a large quantity of T cells from the coculture condition. Second, although RNA and protein samples were collected in parallel with or without treatment, it was not feasible to extract both from the same biological samples, potentially increasing variability. Third, while our multiomics analyses identified important molecular targets, including key immune trafficking nodes such as ICAM1 and ITGB1, ISR/UPR markers (p-eIF2α, ATF4, and CHOP), these findings should be validated with lower-throughput, more targeted assays such as qPCR or Western blotting. Furthermore, a substantial portion of the pathway-level conclusions, including those related to immune trafficking, ISR signaling, and upstream regulator predictions, are derived from IPA and should be interpreted with appropriate caution. Independent experimental validation, such as targeted qPCR, functional immune assays, or gene knockdown and chemical inhibition studies, will be necessary to confirm these predicted pathway activities and minimize overinterpretation of network-level findings. In addition, while the data sets provide extensive insight into T-cell responses, this study does not include transcriptomic or proteomic profiling of melanoma cells, and not all molecular changes could be comprehensively captured within the scope of the current analysis. Finally, studies with in vivo models will be necessary to confirm the current observations. Despite these limitations, our study provides substantial evidence that CBD exerts immunoregulatory effects on T cells and may broaden its potential as an adjunctive immunomodulator.

## 5 Conclusion

In conclusion, this study provides a comprehensive multiomics characterization of CBD’s role in reshaping T-cell function within the melanoma microenvironment through redox- and stress-responsive mechanisms. Using a melanoma-T cell coculture system, we demonstrate that CBD selectively promotes melanoma cell death while inducing coordinated transcriptomic and proteomic remodeling in T cells. Integrated analyses identify modulation of T-cell receptor signaling, translational control via EIF2 signaling, and immune cell trafficking as key outcomes of CBD exposure, with ICAM1 and ITGB1 emerging as central nodes across molecular layers. The observed divergence between transcriptomic and proteomic responses further suggests the importance of stress-adaptive post-transcriptional and translational regulation in shaping immune function. Although additional validation and in vivo studies are warranted, our findings position CBD as a redox-active modulator that engages integrated stress responses to reprogram immune signaling and migratory behavior, providing new insight into the intersection of redox biology and tumor–immune crosstalk.

## Supplementary Material











## Data Availability

The original contributions presented in the study are included in the article and supporting information; further inquiries can be directed to the corresponding authors. Both RNaseq and proteomics raw data sets will be deposited in a public repository upon acceptance of this manuscript.

## Notes

Network analysis results from transcriptomic data (XLS)
Integrated canonical pathway analysis across transcriptomic and proteomic data sets (XLS)
Upstream regulator analysis from transcriptomic data (XLS)
Upstream regulator analysis from proteomic data (XLS)
Integrated multiomics pathway comparison results (XLS)

## Abbreviations

ICIs: immune checkpoint inhibitors
CBD: cannabidiol
DEGs: differentially expressed genes
TCR: T cell receptor
EIF2: eukaryotic initiation factor
ICAM1: intercellular adhesion molecule 1
ITGB1: integrin subunit β 1
PD-1: programmed death 1
CTLA-4: cytotoxic T-lymphocyte-associated antigen 4
FBS: fetal bovine serum
RPMI: roswell park memorial institute
CCK-8: cell counting kit-8
IL-2: interleukin-2
IFN-γ: interferon-γ
PBS: phosphate-buffered saline
DIA: data-independent acquisition
PCT: pressure cycling technology
FDR: false discovery rate
PSM: peptide-spectrum match
PCA: principal component analysis
IPA: ingenuity pathway analysis
SD: standard deviation
